# The Great Mimicker: Zona Zoster at the Mastectomy Site Causing Contralateral Intramammary Lymph Node Enlargement

**DOI:** 10.1155/2012/468576

**Published:** 2012-03-15

**Authors:** Umit Aksoy Ozcan, Evrim Tezcanli, Yesim Yildirim, Melahat Garipagaoglu

**Affiliations:** ^1^Department of Radiology, Acibadem University School of Medicine, Istanbul, Turkey; ^2^Department of Radiation Oncology, Acibadem University School of Medicine, Istanbul, Turkey; ^3^Department of Medical Oncology, Anatolian Health Center Hospital, Istanbul, Turkey

## Abstract

Zona zoster is rarely observed in patients with malignancy; when present, it follows a dermatomal fashion. Involvement of widely separated regions is very rare. Hereby, zona zoster causing enlarged intramammary lymph nodes (IMLN) in the opposite breast is reported for the first time in literature. The masses were hypoechoic on US with no hilum and hypervascular on color Doppler US. MRI showed hypointense masses with type 3 time-intensity curve and adjacent vessel sign. The complete regression of the nodes after the antiviral therapy confirmed the diagnosis. In breast cancer patients, IMLN enlargements may mimic breast cancer metastasis, and zona zoster infection of the mastectomy site may present with contralateral IMLN enlargement due to altered lymphatic drainage. When breast US is not sufficient for the differential diagnosis, breast MRI may warrant proper diagnosis, and prevent unnecessary biopsies. Antiviral treatment with followup would be sufficient for management.

## 1. Introduction

Zona zoster is caused by the varicella zoster virus that remains latent in sensory dorsal root ganglion cells after the first infection or immunization [1]. In immunocompromised patients, the virus travels down the sensory nerve into the skin which is clinically characterized by several groups of painful vesicles situated within the distribution of the spinal sensory nerve [2]. In the classical clinical picture, zona zoster follows a dermatomal fashion. Cases of bilateral involvement and synchronous zosters involving two widely separated regions are rarely reported [2, 3]. Although patients with malignancy are more likely to develop zona zoster, occurrence after breast cancer is rare [4, 5]. To our knowledge, zona zoster of the mastectomy site causes intramammary lymph node (IMLN) enlargement in the opposite breast which has not been reported in the literature so far. Hereby, we present a breast cancer patient who developed zona zoster infection at the mastectomy site, causing IMLN enlargements in the opposite breast, which may be mistaken as breast cancer metastasis.

## 2. Case Report

A 70-year-old breast carcinoma patient was referred for her routine radiological followup. Within the follow-up period the patient had developed painful skin lesions on the mastectomy side in a dermatomal distribution of two-week duration with multiple erythematous firm papulo-nodular lesions arranged along the T-4 segment dermatome, extending from left mammary region to the left scapular region.

The patient had underwent left simple mastectomy and axillary dissection due to breast carcinoma a year ago, and the histopathology revealed moderately differentiated invasive ductal carcinoma sized 2.4 cm, and 21 out of 26 axillary lymph nodes were metastatic. Pathological stage was pT2N3M0. Tumor cells were positive both for estrogen and progesterone receptors and negative for CerbB2. She had received adjuvant chemotherapy consisted of 4 cycles of adriablastin-cyclophosphamide, following 4 cycles of docetaxel and 5000 cGy external radiotherapy to chest wall and regional lymph nodes. Anastrazol was started after radiotherapy.

**Figure 1:**
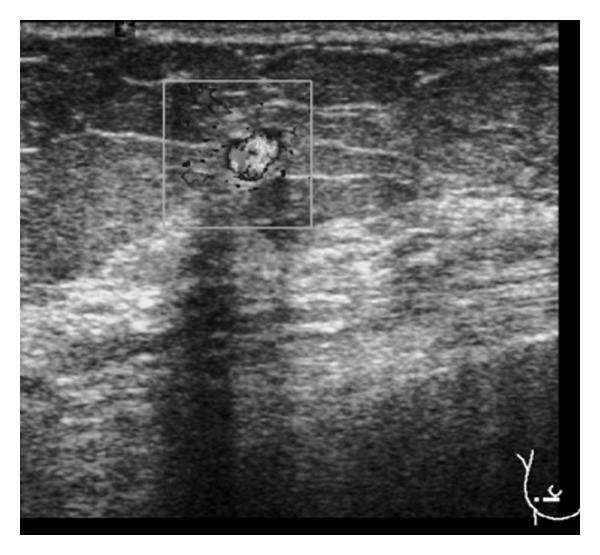
Hypoechoic, mass with lobular contours is observed hypervascular on color Doppler US at the lower outer quadrant at the right breast.

**Figure 2:**
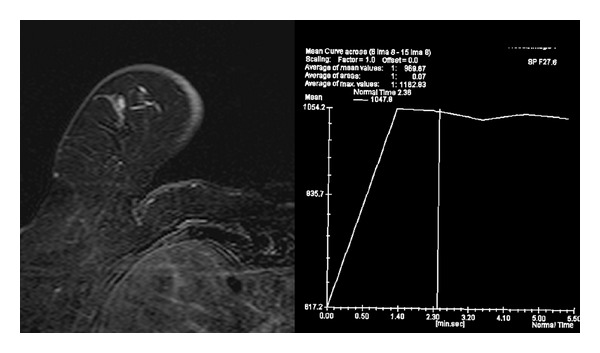
On dynamic series, mass with smooth and lobular margin with an adjacent vessel is observed. Time intensity curve shows rapid washin and minimal washout.

Mammography of the right breast revealed retroareolar fibroglandular densities with no remarkable additional abnormalities. On ultrasound (13–5 MHz wideband multiHertz transducer, Siemens Sonoline Antares, California, USA) 7 mm, 3 mm, and 5 mm hypoechoic, nodular masses with lobular contours were identified at lower outer quadrant near to the areola in the right breast. On color Doppler US, the masses exhibited increased internal and capsular vascularisation (Figure 1). MRI was performed in an open-bore design 1.5T MR unit (Magnetom Espree with Syngo MR B15 software; Siemens, Erlangen, Germany) by using a bilateral 8-channel breast matrix coil. On MRI, the nodules were hypointense on T1- and T2-weighted images, and the margins were smooth and lobular with an adjacent vessel sign. On dynamic series the lesions exhibited homogeneous rapid initial rise over 100%, and minimal washout was observed (Figure 2). Because of extremely intense initial enhancement during the first 1-2 minutes that is well above the usual range for invasive cancers [6], the radiological diagnosis was probable intramammary lymph node enlargement due to zona zoster infection of the mastectomy site.

The patient was started on one-week acyclovir treatment for zona zoster infection. Control US exam after one month showed total regression of the masses, and the patient is symptom-free for 2 years now.

## 3. Discussion

 Our case had shown that, in breast cancer patients, zona zoster may follow an atypical course and present with contralateral enlarged IMLNs. To our knowledge, this is the first reported case of contralateral IMLN enlargement occurring after zona zoster infection of the mastectomy site. The enlargement of IMLNs could mimic metastasis; therefore clinical data is important for the differential diagnosis.

 The reported prevalence of IMLNs is about 2%–28% [7, 8], and they represent a potential extra-axillary site of regional breast cancer metastasis. Similar to the other lymph nodes throughout the body, infections may cause IMLN enlargement. Unlike other infections, typical distribution pattern of the zoster infection is observed along the nerve axis. Contralateral breast is innervated by another dermatome, which is thought to be free of infection. Altered lymphoid drainage after mastectomy may be a possible explanation to the contralateral IMLN enlargement in our case.

Our patient had a history of breast cancer treatment with chemotherapy and external radiotherapy to chest wall and regional lymph nodes. It can be speculated that she was under an immunosuppressed state because of her previous chemotherapy, which may have led to defective cellular immune response and the development of zona zoster. There are some studies reporting higher occurrence of zona zoster ranked 1.9–3.7% in comparison to normal population after receiving radiotherapy [9, 10]. Indeed, part of patients included in these studies received chemotherapy and/or hormone therapy beside radiotherapy. Occurrence of zona zoster after or during chemotherapy was also reported [10]. It was claimed that adjuvant chemotherapy and immunosuppressant could facilitate herpes infection [10]. Other authors highlighted that the location of the lesion was predominantly on the old radiotherapy side [5, 9]. In present case, zona zoster lesion was located on the breast cancer side, and enlarged lymph nodes were located in the opposite site of the cancer. Enlarged lymph nodes may mimic malignant disease as in our case, which could be primary tumor metastasis or related to second primary. In both conditions patient prognosis and treatment is affected. Of the patients with IMLN identified, 10% contain metastatic disease [8]. Early diagnosis of recurrence is important for disease control.

In the absence of classical fatty hilum and regular ovoid cortex, radiological differential diagnosis of enlarged IMLNs is not always possible. Mammography fails to identify over half of the IMLNs visualized on sonography [11]. Detection of metastatic IMLNs via breast imaging is focused on enlargement of the nodes to 1 cm, and absence of the central echogenic/fatty hilum [11, 12]. In our case the detected masses were smaller than 1 cm and the central hilum was not present. Detection based on these US characteristics has become insufficient as advances in technology demonstrate additional characteristic for further differentiation of smaller masses. The majority of the metastatic IMLNs are reported to have an eccentrically displaced echogenic hilum with a ratio of its volume to the total volume of the lymph node of less than 50%, thickening of the nodal cortex, and pronounced hypoechogenicity of the nodal cortex [11, 13]. However, Edeiken-Monroe et al. [11] reported that, contrary to the description in the literature, in their series most of the nodes retained an oval appearance with lobular margins and no significant distal sound modulation as in our case.

 On the morphologic analysis of MRI, the margins of the observed masses in the presented case were smooth, and lobular, which is associated with a 90–95% negative predictive value for carcinoma [14–16]. On the contrary, the intensity versus time curve showed fast enhancement with washout, unlike benign lesions, which tend to have a gradual increase in enhancement. Various benign conditions which produce contrast enhancement patterns that are hard to distinguish from malignant processes may mimic malignant lesions [14]. Nevertheless, 10%–20% of breast malignancies are well-circumscribed masses [16]. Adjacent vessel sign is an MRI sign indicative of malignancy and was observed in our case [17, 18]. Breast MRI plays a substantial role in distinguishing between well-circumscribed benign and malignant breast lesions. Mass lesions with washout on dynamic series are generally considered malignant in a patient with known malignancy [6]. However, extremely intense initial enhancement during the first 1-2 minutes that is well above the usual range for invasive cancers is not typical of carcinomas [6]. This finding was consistent with our findings in this paper, and the masses were considered as enlarged IMLNs caused by atypical zona zoster infection of the contralateral mastectomy site. Cancers are less likely to show this high initial peak [6]. The enhancement pattern of IMLNs in dynamic MRI series may mimic malignant lesions with rapid initial increase and washout kinetics [6]. The complete regression of the IMLNs after the antiviral therapy confirmed our initial diagnosis.

In conclusion, in breast cancer patients, zona zoster infection may present with atypical findings and cause IMLN enlargement on the opposite side. When breast US is not sufficient for the differential diagnosis, further assessment with breast MRI may warrant proper diagnosis of IMLN enlargement. This would prevent unnecessary biopsies, and antiviral treatment with followup would be sufficient for management.
